# Racial disparities in antibiotic selection for community-acquired pneumonia in hospitalized patients

**DOI:** 10.1017/ice.2025.10371

**Published:** 2026-03

**Authors:** Ramara E. Walker, Rebecca Schulte, Andrea M. Pallotta, Ming Wang, Abhishek Deshpande, Michael Rothberg

**Affiliations:** 1 Department of Pharmacy, Cleveland Clinic, Cleveland, OH, USA; 2 Department of Quantitative Health Sciences, Cleveland Clinic, Cleveland, OH, USA; 3 Department of Population and Quantitative Health Sciences, Case Western Reserve University, Cleveland, OH, USA; 4 Center for Value-Based Care Research, Primary Care Institute, Cleveland Clinic, Cleveland, OH, USA; 5 Alice L. Walton School of Medicine, Bentonville, AR, USA

## Abstract

**Objective:**

Community-acquired pneumonia (CAP) is a leading cause of hospitalization and mortality in the US. Studies report racial disparities in various infectious syndromes. Our objective was to assess the relationship between patient race and antibiotic prescribing in inpatient CAP management.

**Design:**

Retrospective cohort study.

**Setting:**

11 Cleveland Clinic community hospitals.

**Patients:**

Patients aged ≥18 years hospitalized with CAP between November 1, 2022, and January 31, 2025.

**Methods:**

Parametric and non-parametric methods were used to describe demographic and clinical differences by race. The association between race and extended spectrum antibiotic (ESA) guideline concordance was assessed using multivariable logistic regression models adjusting for age, gender, admission source, area deprivation index (ADI), hospital, diabetes, cardiovascular disease, chronic respiratory disease, renal failure, liver disease, immunocompromising condition, alcohol and substance use disorder, dialysis, and clinical instability and severity on day 1.

**Results:**

In bivariate analyses, Non-Hispanic Black (NHB) patients were less likely than NHW patients to receive ESA guideline-concordant CAP therapy (63.2% vs 64.4%; OR = 0.91, *P* = .2). After adjusting for patient characteristics, there were no differences between NHB and NHW patients in receipt of ESA therapy (adjusted OR = 0.93; 95% CI = 0.83, 1.00). After adjusting for hospital, NHB patients were more likely to receive ESA guideline-concordant CAP therapy (adjusted OR = 1.17; 95% CI = 1.06, 1.30).

**Conclusion:**

NHB patients were more likely to receive ESA-guideline concordant therapy, but this was influenced by where they sought care. Further studies are needed to understand why prescribing varies across hospitals.

## Introduction

Community-acquired pneumonia (CAP) is one of the most common causes of hospitalization and mortality in the United States.^1^ There have been racial disparities observed in CAP pertaining to incidence, severity, treatment, and outcomes which pose a public health concern.^2,3^ Limited, conflicting data exist regarding racial disparities in antimicrobial use and antimicrobial resistance.^4–7^ A few studies have suggested that Black, Hispanic, and lower-income people are at increased risk of community-acquired drug-resistant pathogens such as methicillin-resistant *Staphylococcus aureus* (MRSA) from a typically sterile body site (e.g., blood, cerebrospinal fluid, and internal body fluid) and drug-resistant *Streptococcus pneumoniae* CAP.^8,9^ However, socioeconomic factors explained these observed differences—there was no racial predisposition to harboring resistant pathogens after adjusting for confounders.^8,9^ Given the paucity of literature evaluating whether racial disparities exist in treatment selection for patients hospitalized with CAP—particularly as most existing data on antimicrobial stewardship disparities focus on the outpatient setting—additional studies are needed.^10^ The aim of this study was to describe racial differences in antibiotic prescribing for patients hospitalized with CAP.

## Methods

### Sample

We conducted a retrospective cohort study across 11 Cleveland Clinic community hospitals. We included patients ≥18 years of age who were admitted with a diagnosis of CAP from November 1, 2022, to January 31, 2025. CAP cases were identified using ICD-10 diagnostic codes, and we also included patients for whom clinicians documented CAP in free-text diagnosis fields. Although a patient may have been admitted multiple times during the study period, we considered each admission as an independent observation. Patients were excluded if they had a diagnosis of cystic fibrosis, were discharged from an acute care hospital within the last 7 days, had hospital-acquired pneumonia (HAP) indicated in their problem list, were admitted to the intensive care unit within 24 hours, had comfort care measures, had any bacterial pathogen isolated in blood or respiratory cultures 72 hours prior to admission, transferred from another hospital, or had an unknown race or ethnicity. This study was approved by Cleveland Clinic’s Institutional Review Board.

### Measures

The primary predictor variable was patient race, which was self-reported. Comparisons were limited to non-Hispanic Black (NHB) and non-Hispanic White (NHW) patients due to small sample sizes and heterogeneity among patients from other racial and ethnic groups, which precluded statistically reliable subgroup analyses. Therefore, these other groups were excluded from the analysis. Race was conceptualized and used as a social, rather than biological, variable to reflect the influence of structural and interpersonal factors on healthcare delivery.^11^

The primary outcome was extended-spectrum antibiotic (ESA) guideline-concordant CAP therapy. This encompassed both patients for whom ESA therapy was recommended and who received it, as well as those for whom ESA therapy was not indicated and who appropriately did not receive it. ESA guideline concordance was based on the 2019 IDSA/ATS guidelines^12^ whereas clinical severity was defined by the 2007 IDSA/ATS CAP severity criteria guidelines.^13^ Treatment of non-severe pneumonia was considered guideline-concordant if it included a beta-lactam plus a macrolide or respiratory fluoroquinolone with the addition of or escalation to MRSA or *Pseudomonas aeruginosa* coverage only in the event of prior respiratory isolation of these organisms in the past year or in response to a positive blood culture. Conversely, treatment of severe pneumonia was considered guideline-concordant if it included a beta-lactam plus a macrolide or a beta-lactam plus a respiratory fluoroquinolone with the addition of MRSA and/or *Pseudomonas* coverage in the setting of prior respiratory culture isolation within the past year or recent hospitalization with IV antibiotics in the past 90 days. We defined non-extended spectrum antibiotics (non-ESA) as ampicillin/sulbactam, amoxicillin/clavulanate, ceftriaxone, cefdinir, cefuroxime, cefpodoxime, azithromycin, doxycycline, levofloxacin, and moxifloxacin. All other antibiotics targeting CAP treatment were defined as ESA.

Demographic and clinical characteristics were collected for all patients. Clinical variables, including indicators of clinical stability, disease severity, CURB-65 scores, and ventilatory support were assessed within the first 24 hours of hospital admission and were derived from the minimum and maximum recorded values during that period.

### Statistical analysis

Normally distributed variables were summarized as mean and standard deviation (SD); non-normally distributed variables as median and interquartile range (IQR). Categorical variables were summarized as counts and percentages. Parametric and non-parametric methods were used to compare demographic and clinical differences by race. Rates of concordant ESA therapy were compared across racial groups and hospitals. The association between racial group and guideline concordance was assessed using two multivariable logistic regression models adjusting for age, gender, ADI, and comorbidities (diabetes, cardiovascular disease, chronic respiratory disease, renal failure, dialysis, liver disease, immunocompromising condition, alcohol use disorder, substance use disorder), clinical instability on day 1, and American Thoracic Society (ATS) severity. Variables were selected based on bivariate associations, literature, and clinical judgment regarding anticipated impact on guideline concordance. The first model did not adjust for hospital; the second did. Because < 1% of our sample was missing any data (ATS severity), we conducted a complete case analysis rather than using other adjustment techniques. Tests were two-sided with a significance level of 0.05. Analyses were conducted using R version 4.4.0.

## Results

From November 1, 2022, to January 31, 2025, there were 14,991 eligible patient admissions included in the sample. There were 13,091 unique patients, with 89% of patients having one eligible encounter. Table 1 describes the sample population by race. Compared to NHW, NHB were more likely to be younger, female, live in a higher area of deprivation, and have Medicaid insurance. They were also more likely to have diabetes, renal failure, alcohol and substance use disorder, and be clinically stable on day 1, but less likely to have an immunocompromising condition. No significant differences were observed amongst groups regarding resistant organism isolation.

**Table 1.** Demographic, clinical, and hospitalization characteristics of admissions for community-acquired pneumonia, stratified by race

Characteristic	Overall admissions, *N* = 16,258	Non-Hispanic White, *N* = 11,521	Non-Hispanic Black, *N* = 3,470	Other minoritized,*N* = 1,267	*P*-value
Age (years)	75 [64–84]	77 [67 – 85]	67 [56–77]	72 [56–82]	< .001
Gender					< .001
Female	8,371 (51%)	5,848 (51%)	1,890 (54%)	633 (50%)	
ADI					< .001
Low: 1–3	5,923 (36%)	5,094 (44%)	390 (11%)	439 (35%)	
Medium: 4–6	3,981 (24%)	3,138 (27%)	588 (17%)	255 (20%)	
High: 7–10	5,490 (34%)	2,695 (23%)	2,313 (67%)	482 (38%)	
Unknown	864 (5.3%)	594 (5.2%)	179 (5.2%)	91 (7.2%)	
Insurance					< .001
Private	3,546 (22%)	2,581 (22%)	672 (19%)	293 (23%)	
Medicare	10,529 (65%)	7,993 (69%)	1,846 (53%)	690 (54%)	
Medicaid	1,728 (11%)	707 (6.1%)	824 (24%)	197 (16%)	
Other/unknown	455 (2.8%)	240 (2.1%)	128 (3.7%)	87 (6.9%)	
Admission source					< .001
Community	15,954 (98%)	11,274 (98%)	3,422 (99%)	1,258 (99%)	
SNF/ICF	277 (1.7%)	227 (2.0%)	43 (1.2%)	7 (0.6%)	
Other	27 (0.2%)	20 (0.2%)	5 (0.1%)	2 (0.2%)	
Selected comorbidities					
Diabetes	5,696 (35%)	3,640 (32%)	1,517 (44%)	539 (43%)	< .001
Cardiovascular disease	13,003 (80%)	9,253 (80%)	2,827 (81%)	923 (73%)	< .001
Chronic respiratory disease	9,506 (58%)	6,817 (59%)	2,037 (59%)	652 (51%)	< .001
Renal failure	3,519 (22%)	2,270 (20%)	1,000 (29%)	249 (20%)	< .001
Liver disease	2,194 (13%)	1,520 (13%)	481 (14%)	193 (15%)	.1
Immunocompromising condition	4,892 (30%)	3,816 (33%)	773 (22%)	303 (24%)	< .001
Alcohol use disorder	1,336 (8.2%)	869 (7.5%)	405 (12%)	62 (4.9%)	< .001
Substance use disorder	2,009 (12%)	1,150 (10.0%)	728 (21%)	131 (10%)	< .001
Pathogen detected					.004
None detected	12,243 (75%)	8,597 (75%)	2,686 (77%)	960 (76%)	
Detected	4,015 (25%)	2,924 (25%)	784 (23%)	307 (24%)	
Pathogen type*					.011
Atypical bacterial	182 (4.5%)	116 (4.0%)	53 (6.8%)	13 (4.2%)	
Non-Atypical bacterial pathogen	1,514 (38%)	1,095 (37%)	321 (41%)	98 (32%)	
MRSA	45 (1.1%)	32 (1.1%)	10 (1.3%)	3 (1.0%)	
*Pseudomonas*	189 (4.7%)	142 (4.9%)	33 (4.2%)	14 (4.6%)	
Viral pathogen	1,559 (39%)	1,149 (39%)	273 (35%)	137 (45%)	
More than one pathogen	526 (13%)	390 (13%)	94 (12%)	42 (14%)	
Resistant bacterial organism					.4
Non-resistant	1,929 (79%)	1,383 (78%)	413 (81%)	133 (78%)	
Resistant	527 (21%)	392 (22%)	98 (19%)	37 (22%)	
CURB65	2 [1–3]	2 [1–3]	1 [1–2]	2 [1–2]	< .001
Clinical severity (ATS 2007 criteria)	392 (2.4%)	285 (2.5%)	82 (2.4%)	25 (2.0%)	.6
Unknown	165	109	36	20	
Clinical stability on day 1					.026
Stable	8,310 (51%)	5,856 (51%)	1,835 (53%)	619 (49%)	
Unstable	7,948 (49%)	5,665 (49%)	1,635 (47%)	648 (51%)	
Invasive mechanical ventilation	15 (<0.1%)	7 (<0.1%)	7 (0.2%)	1 (<0.1%)	.06
Unknown	167	109	38	20	
Non-invasive ventilation	873 (5.4%)	656 (5.7%)	151 (4.4%)	66 (5.2%)	.009
Dialysis	629 (3.9%)	273 (2.4%)	305 (8.8%)	51 (4.0%)	< .001
Hospital					< .001
1	1,979 (12%)	1,467 (13%)	452 (13%)	60 (4.7%)	
2	1,891 (12%)	1,660 (14%)	102 (2.9%)	129 (10%)	
3	748 (4.6%)	244 (2.1%)	493 (14%)	11 (0.9%)	
4	2,388 (15%)	1,853 (16%)	265 (7.6%)	270 (21%)	
5	2,062 (13%)	1,637 (14%)	367 (11%)	58 (4.6%)	
6	1,991 (12%)	1,740 (15%)	149 (4.3%)	102 (8.1%)	
7	508 (3.1%)	236 (2.0%)	153 (4.4%)	119 (9.4%)	
8	1,262 (7.8%)	710 (6.2%)	521 (15%)	31 (2.4%)	
9	1,135 (7.0%)	1,077 (9.3%)	38 (1.1%)	20 (1.6%)	
10	983 (6.0%)	192 (1.7%)	776 (22%)	15 (1.2%)	
11	1,311 (8.1%)	705 (6.1%)	154 (4.4%)	452 (36%)	

Note. IQR, Interquartile Range; ADI, Area Deprivation Index; SNF, Skilled Nursing Facility; ICF, Intermediate Care Facility; ATS, American Thoracic Society; CAP, Community-Acquired Pneumonia; ESA, Extended Spectrum Antibiotic; MRSA, Methicillin-resistant Staphylococcus aureus.

*Categories are mutually exclusive. Data presented as *n* (%) for categorical variables and median [IQR] for continuous variables, unless otherwise specified.

**Figure 1.**
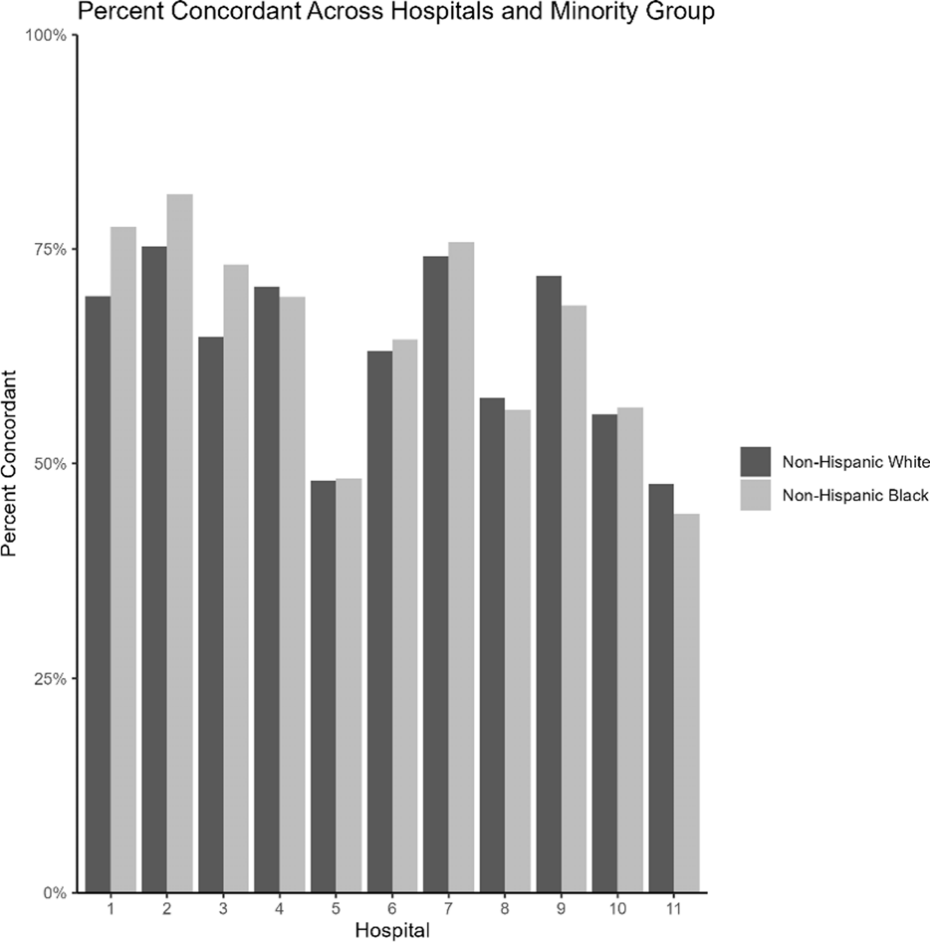
Percent of concordant cap therapy across hospitals and racial group.

The percentage of NHW patients varied across the 11 hospitals from 19.8% to 96.6% (Supplemental Table 1). Overall, 413 (2.8%) of patients met the current guidelines for ESA (Table 2). Among those recommended to receive ESA per guidelines, 72.4% received an ESA within 24 hours. Of those not recommended to receive ESA per guidelines, 36.1% received an ESA within 24 hours. The primary outcome, guideline concordance of ESA, was moderate to moderately high. Specifically, concordance was 64.4% in NHW patients and 63.2% in NHB patients (*P* = .2), while hospital-level concordance ranged widely from 44% to 82% (Figure 1). NHB patients were more likely than NHW patients to receive ESA without a documented indication (37.2% vs 35.7%; *P* = .121), indicating potential overuse. Furthermore, ESA prescribing with an appropriate indication was more common among NHB patients compared to NHW patients, although this difference did not reach statistical significance (78.8% vs 70.4%; *P* = .133) (Table 2).

**Table 2.** Patterns of extended-spectrum antibiotic (ESA) use by race among hospital admissions for community-acquired pneumonia

Antibiotic use pattern	Overall admissions *N* = 14,991	Non-Hispanic White *N* = 11,521	Non-Hispanic Black *N* = 3,470	*P*-Value
Recommended ESA per guidelines, *n* (%)	413 (2.8%)	314 (2.7%)	99 (2.9%)	.7
Received ESA (concordant)	299 (72.4%)	221 (70.4%)	78 (78.8%)	.13
Did not receive ESA (discordant)	114 (27.6%)	93 (29.6%)	21 (21.2%)	.13
Not recommended ESA per guidelines, *n* (%)	14,578 (97.2%)	11,207 (97.3%)	3,371 (97.1%)	.7
Received ESA (discordant)	5,261 (36.1%)	4,006 (35.7%)	1,255 (37.2%)	.12
Did not receive ESA (concordant)	9,317 (63.9%)	7,201 (64.3%)	2,116 (62.8%)	.12

Note. ESA, Extended Spectrum Antibiotic. Data presented as *n* (%) for categorical variables.

Multivariable logistic regression was performed to evaluate the association between race and ESA guideline-concordant CAP therapy, with and without adjustment for hospital. Adjusting for patient-level characteristics had no impact on the odds of receiving ESA guideline-concordant therapy (adjusted OR, 0.91; 95% CI [0.83, 1.00]). After adjusting for hospital, NHB patients were more likely to receive ESA therapy concordant with CAP guidelines (adjusted OR, 1.17; 95% CI [1.06, 1.3]). Hospital-specific guideline concordance is presented in Supplemental Figure 1, with adjusted odds ratios ranging from 0.85 to 1.63 with wide confidence intervals.

## Discussion

This study assessed the association between race and ESA prescribing in hospitalized patients with CAP and found that NHB patients were more likely to receive ESA therapy consistent with CAP guidelines after accounting for hospital-level variation, despite no difference observed after adjusting for patient-level characteristics. We found no difference in the probability of a resistant organism isolated during the current hospital admission or severity of illness—two factors that might be expected to increase the use of ESA—based on race. These findings indicate that differences in CAP treatment are largely attributable to variation in where minoritized and non-minoritized patients receive care.

A hospital’s access to resources and its system-level process of care can be an important driver of disparities. Hausmann and colleagues sought to examine within and between-hospital racial/ethnic disparities in quality indicators and mortality for hospitalized patients with pneumonia and found that Black and Hispanic patients were significantly more likely to be treated at urban and large/teaching hospitals and hospitals with diverse patient populations than White patients.^14^ Moreover, they demonstrated that patients receiving care at hospitals serving higher concentrations of Black and Hispanic patients had lower odds of receiving 7 of 8 recommended process quality indicators which included appropriate antibiotic selection within 24 hours of admission.^14^ Similarly, Jha and colleagues identified that hospitals with either a higher volume or proportion of Black patients were most often large, urban teaching hospitals and provided lower quality of care for pneumonia than hospitals with a low volume of Black patients.^15^

Historic racialized housing and zoning policies, including redlining, have shaped residential patterns and constrained healthcare access for minoritized populations, often channeling care toward hospitals serving their immediate communities.^16^ They may also not be aware of differences in care quality, especially if they are not easily identified. Efforts to reduce these disparities should therefore target overall performance at hospitals with large minority patient populations.

We found that in slightly more than half of hospitals within our system, NHB patients were more likely than NHW patients to receive guideline-concordant antibiotics; however, these hospitals served primarily NHW patients suggesting that hospital-level factors may be more influential than prescriber bias. The current evidence is mixed related to within-hospital variation of the impact on race on CAP-guideline-concordant care. Two studies found within-hospital racial disparities in CAP management which included measures such as receipt of pneumococcal and influenza vaccinations, smoking cessation counseling, initial antibiotic timing, and oxygenation assessment.^14,15^ Another study found no racial differences in the receipt of CAP-guideline-concordant antibiotics between Black and White patients aged ≥65 years, regardless of admission to a medical ward or intensive care unit within the Veterans Health Administration.^17^ Similar to our findings, Hasnain-Wynia and colleagues found that almost half of the racial/ethnic disparities for oxygenation assessment for CAP and antibiotic therapy within 8 hours of arrival for CAP reflected between-hospital rather than within-hospital factors.^18^ Taken together, these findings suggest that quality improvement initiatives including antimicrobial stewardship efforts should target hospitals with higher concentrations of minority patients, and further research should seek to understand the association between where minoritized patients seek care and the quality of care that they receive.

A key strength of this study is its focus on inpatient management of CAP, an area where literature on racial and ethnic disparities remains limited compared to outpatient care. This investigation reflects real-world conditions, where antibiotic prescribing decisions were made independently by clinicians without protocolized intervention. The study included a diverse mix of hospital settings (e.g., urban teaching hospitals and smaller community hospitals) and clinician types (e.g., hospital-employed vs private-practice providers) within a single health system, enhancing generalizability.

There are several limitations. First, our analysis compared only NHB and NHW patients; we were unable to evaluate other racial or ethnic minority groups due to small sample sizes and heterogeneity. Second, we lacked data on factors influencing hospital choice, limiting assessment of potential selection biases. Third, CAP cases were identified using ICD-10 codes and provider-entered free-text diagnoses, which may introduce misclassification. Finally, we were unable to directly measure prescriber bias or reasons for hospital-level variation in prescribing. The lack of racial differences in prescribing within hospitals suggests that such bias, if present, may not be a major driver. Nonetheless, residual confounding by unmeasured factors cannot be excluded, underscoring the need for further research.

## Conclusion

Overall, among patients hospitalized with CAP, NHB patients were more likely than NHW patients to receive ESA guideline-concordant care. This difference appears to be driven by hospital-level factors rather than individual patient characteristics. Future research should explore the underlying reasons for interhospital variation in prescribing practices to support more equitable and effective antibiotic stewardship.

## Supporting information

Walker et al. supplementary material Walker et al. supplementary material

## Supplementary material

The supplementary material for this article can be found at https://doi.org/10.1017/ice.2025.10371.
