# Supplementary material for: Racial disparities in antibiotic selection for community-acquired pneumonia in hospitalized patients
Source: Infect Control Hosp Epidemiol. 2025 Dec 9;47(3):305–10. doi: 10.1017/ice.2025.10371 (PMC12932918; doi:10.1017/ice.2025.10371)
Supplement: Walker et al. supplementary material [file S0899823X25103711sup001.docx]

**Supplemental Figure 1. Association between Non-Hispanic Black Race and Guideline Concordance for Patients with Community-Acquired Pneumonia by Hospital**

**
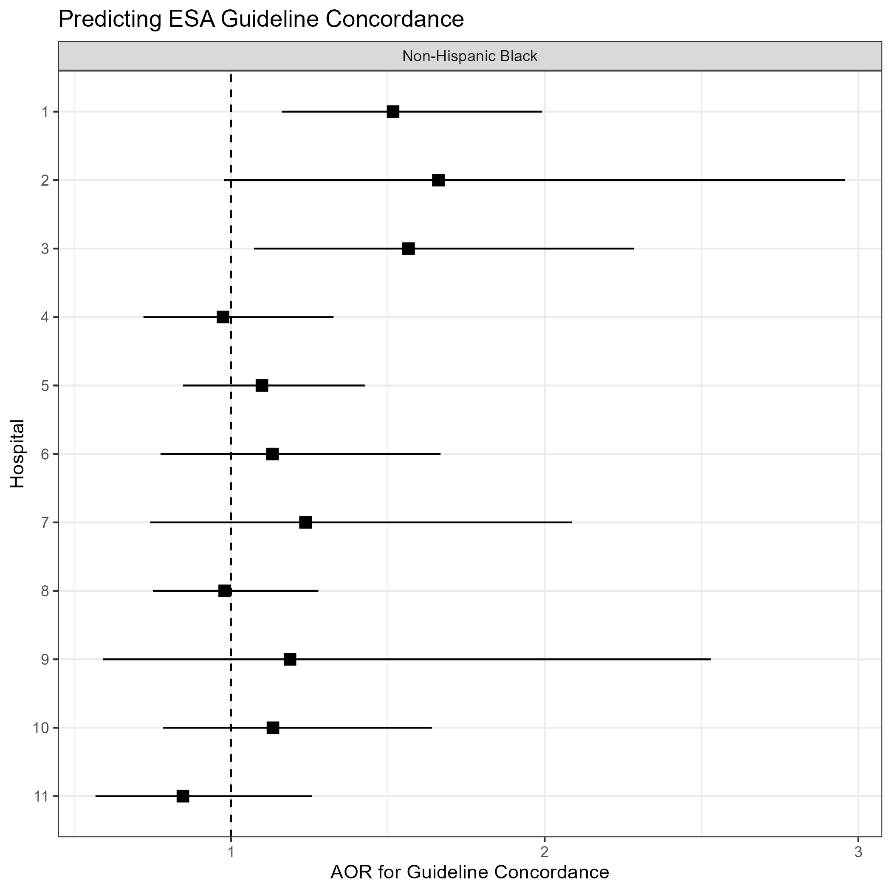
**

**Supplemental Table 1. Hospital Characteristics**

| Hospital | Eligible Admissions | Non-Hispanic White | Non-Hispanic Black | Number of Beds | Teaching Hospital |
| --- | --- | --- | --- | --- | --- |
| 1 | 1919 | 1467 (76.4%) | 452 (23.6%) | 532 | Teaching Hospital |
| 2 | 1762 | 1660 (94.2%) | 102 (5.8%) | 126 | Non-Teaching Hospital |
| 3 | 737 | 244 (33.1%) | 493 (66.9%) | 110 | Non-Teaching Hospital |
| 4 | 2118 | 1853 (87.5%) | 265 (12.5%) | 488 | Teaching Hospital |
| 5 | 2004 | 1637 (81.7%) | 367 (18.3%) | 341 | Non-Teaching Hospital |
| 6 | 1889 | 1740 (92.1%) | 149 (7.9%) | 332 | Non-Teaching Hospital |
| 7 | 389 | 236 (60.7%) | 153 (39.3%) | 119 | Teaching Hospital |
| 8 | 1231 | 710 (57.7%) | 521 (42.3%) | 195 | Non-Teaching Hospital |
| 9 | 1115 | 1077 (96.6%) | 38 (3.4%) | 148 | Non-Teaching Hospital |
| 10 | 968 | 192 (19.8%) | 776 (80.2%) | 167 | Teaching Hospital |
| 11 | 859 | 705 (82.1%) | 154 (17.9%) | 230 | Teaching Hospital |
